# Complete radiographic response to immunotherapy in a patient with metastatic anaplastic thyroid cancer – a case report and review of the literature

**DOI:** 10.3389/fonc.2026.1780838

**Published:** 2026-06-02

**Authors:** Monika Mierzejewska, Lin Akily, William Karlsen, Jacek Teodorczyk, Bartłomiej Tomasik, Jacek Rutkowski, Piotr Wiśniewski, Maciej Śledziński, Karolina Markiet, Jolanta Szade, Renata Zaucha, Wojciech Cytawa

**Affiliations:** 1 Department of Nuclear Medicine, Medical University of Gdańsk, Gdańsk, Poland; 2 Department of Internal Medicine, Ljungby Hospital, Ljungby, Sweden; 3 Department of Internal Medicine, Visby Hospital, Visby, Sweden; 4 Department of Oncology and Radiotherapy, Medical University of Gdańsk, Gdańsk, Poland; 5 Centre for Experimental Cardiooncology, Medical University of Gdańsk, Gdańsk, Poland; 6 Department of Endocrinology and Internal Medicine, Medical University of Gdańsk, Gdańsk, Poland; 7 Department of General, Endocrine and Transplant Surgery, Medical University of Gdańsk, Gdańsk, Poland; 8 2nd Division of Radiology, Medical University of Gdańsk, Gdańsk, Poland; 9 Department of Pathomorphology, Medical University of Gdańsk, Gdańsk, Poland

**Keywords:** anaplastic thyroid cancer, complete response, immune checkpoint inhibitor, immune-related adverse event, immunotherapy

## Abstract

Here we present the case of a 76-year-old female with anaplastic thyroid cancer (ATC), stage pT3bN0, who was initially treated with total thyroidectomy followed by adjuvant radiotherapy after non-radical (R1) surgery. Three months after initial treatment, the disease rapidly progressed and disseminated to the lungs, causing significant pulmonary insufficiency. Due to high PD-L1 expression of the tumor, the patient was offered off-label immunotherapy with an immune checkpoint inhibitor (ICI) (nivolumab monotherapy) and demonstrated a remarkable radiographic and clinical response, achieving prolonged progression-free survival (12 months) and overall survival (18 months). ICIs may represent a valuable therapeutic option for patients with metastatic ATC exhibiting high PD-L1 expression. The presented case report and literature review underscore the need for clinical trials to confirm the efficacy of immunotherapy in ATC.

## Introduction

Immunotherapy has revolutionized modern oncology. Remarkable response rates and survival benefits have been reported with immune checkpoint inhibitors (ICI) across a range of solid tumors at the metastatic stage. Anaplastic thyroid cancer (ATC) is rare, yet it is among the most aggressive malignancies, with very limited therapeutic options once the disease has disseminated. To date, only a small number of publications have reported on ICI treatment in ATC, most of them being single case reports (**Table 1**; **Supplementary Material**) (1–29). Moreover, data on the use of ICIs without combined targeted therapy remain particularly scarce.

The aim of this article is to present the case of a patient with recurrent metastatic ATC, who, after exhausting standard therapeutic options, was treated with ICI-based immunotherapy, and subsequently achieved a complete radiological and clinical response of multiple lung metastases.

## Case presentation

**Figure 1:**
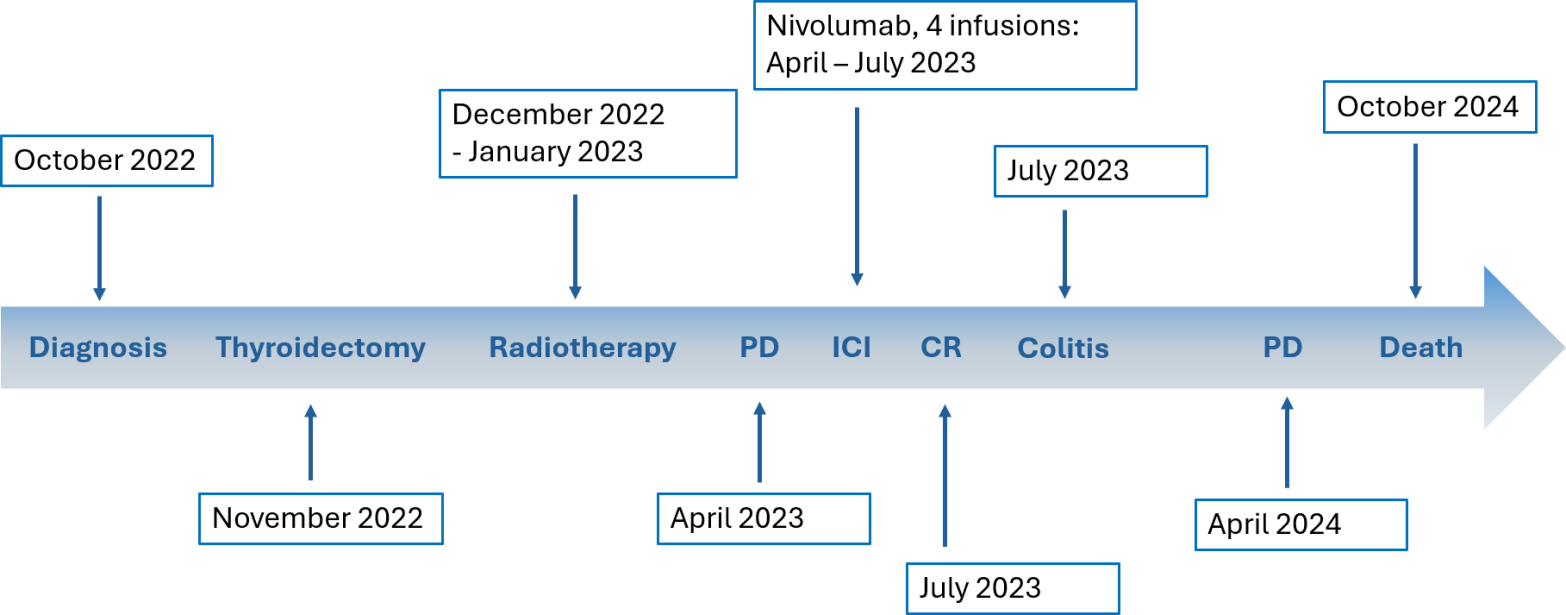
Chronological summary of patient’s history, diagnoses, treatments, and outcomes. PD, progressive disease; ICI, immune checkpoint inhibitor.

A 76-year-old female presented to her primary care physician in October 2022 (**Figure 1**) with a complaint of a neck lump that had been palpable for two weeks. Initially, the lesion was accompanied by dysphagia, following an upper respiratory tract infection. The medical history revealed a 20-year follow-up for nodular goiter, including a right thyroid lobe nodule that underwent fine-needle aspiration biopsy (FNAB) in 2003 and 2007, both confirming its benign character.

**Figure 2:**
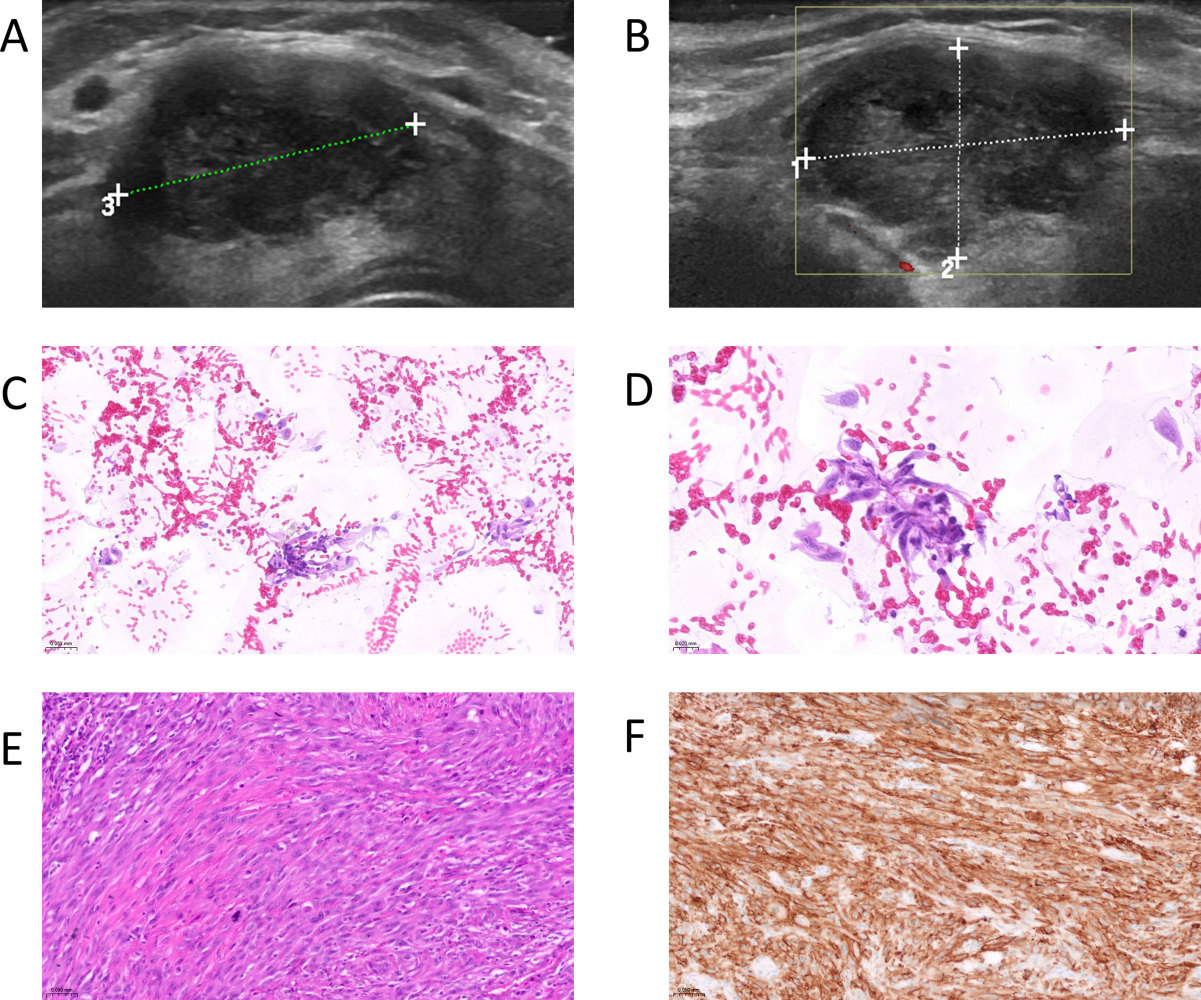
Ultrasound image of the primary thyroid lesion at diagnosis. A markedly hypoechoic, heterogeneous area measuring 24 × 16 × 22 mm with irregular margins is visible in the isthmus [**(A)**, transverse view], showing no intralesional vascular signal [**(B)**, longitudinal view with Power Doppler]. At this stage, the finding could have been linked with either a conglomerate of thyroid nodules or a prior hemorrhagic event within the pyramidal lobe nodule, the latter being supported by the patient’s report of a palpable reduction in lesion size. Fine-needle aspiration cytology revealed atypical, partially spindle-shaped cells with nuclear atypia, consistent with malignant cells, most likely anaplastic thyroid carcinoma, Bethesda System Category VI [**(C)**, ×20; **(D)**, ×40]. Postoperative histopathological examination of hematoxylin and eosin-stained sections confirmed undifferentiated/anaplastic follicular cell-derived thyroid carcinoma, spindle cell subtype **(E)**. Immunohistochemical staining showed high expression of PD-L1 on cancer cells: TPS 99%, CPS 100 **(F)**.

Physical examination demonstrated a palpable, non-tender, poorly mobile, hard-consistency mass measuring approximately 3 cm, located in the region of the pyramidal lobe, slightly to the right of the midline. Neck ultrasound examination revealed a hypoechoic, polycyclic structure measuring 25x16x22 mm with poor vascularization (**Figures 2A, B**). Soon after, a FNAB identified cancer cells, most likely of anaplastic origin (category VI according to the 2017 Bethesda System for Reporting Thyroid Cytopathology; **Figures 2C, D**). Computed tomography (CT) of the neck and thorax showed no signs of metastases, therefore the patient was eligible for total thyroidectomy with central neck dissection (CND). Postoperative histopathological examination revealed poorly differentiated/anaplastic thyroid cancer, pT3bN0, stage IVB according to the 8th edition of the American Joint Committee on Cancer (AJCC) TNM classification system (**Figure 2E**). Due to a non-radical (R1) resection, the patient underwent radical radiotherapy to three target volumes between December 2022 and January 2023. Clinical target volume 1 (CTV1) encompassed the area of microscopic residual disease (R1) with an approximately 5 mm margin; CTV2 covered the thyroidectomy bed; and CTV3 included the bilateral cervical lymphatic drainage regions at nodal levels II–V, level VI, and the upper mediastinum. An isotropic margin of 3 mm was applied to generate the planning target volumes 1 to 3 (PTV 1-3). The prescribed doses to PTV1, PTV2, and PTV3 were 50 Gy in 25 fractions, 60 Gy in 30 fractions, and 66 Gy in 33 fractions, respectively. At the end of radiotherapy, the patient reported typical irritation and redness of the chest and neck skin, sore throat, and excessive mucous secretion in the upper respiratory tract, which resolved after standard supportive care.

**Figure 3:**
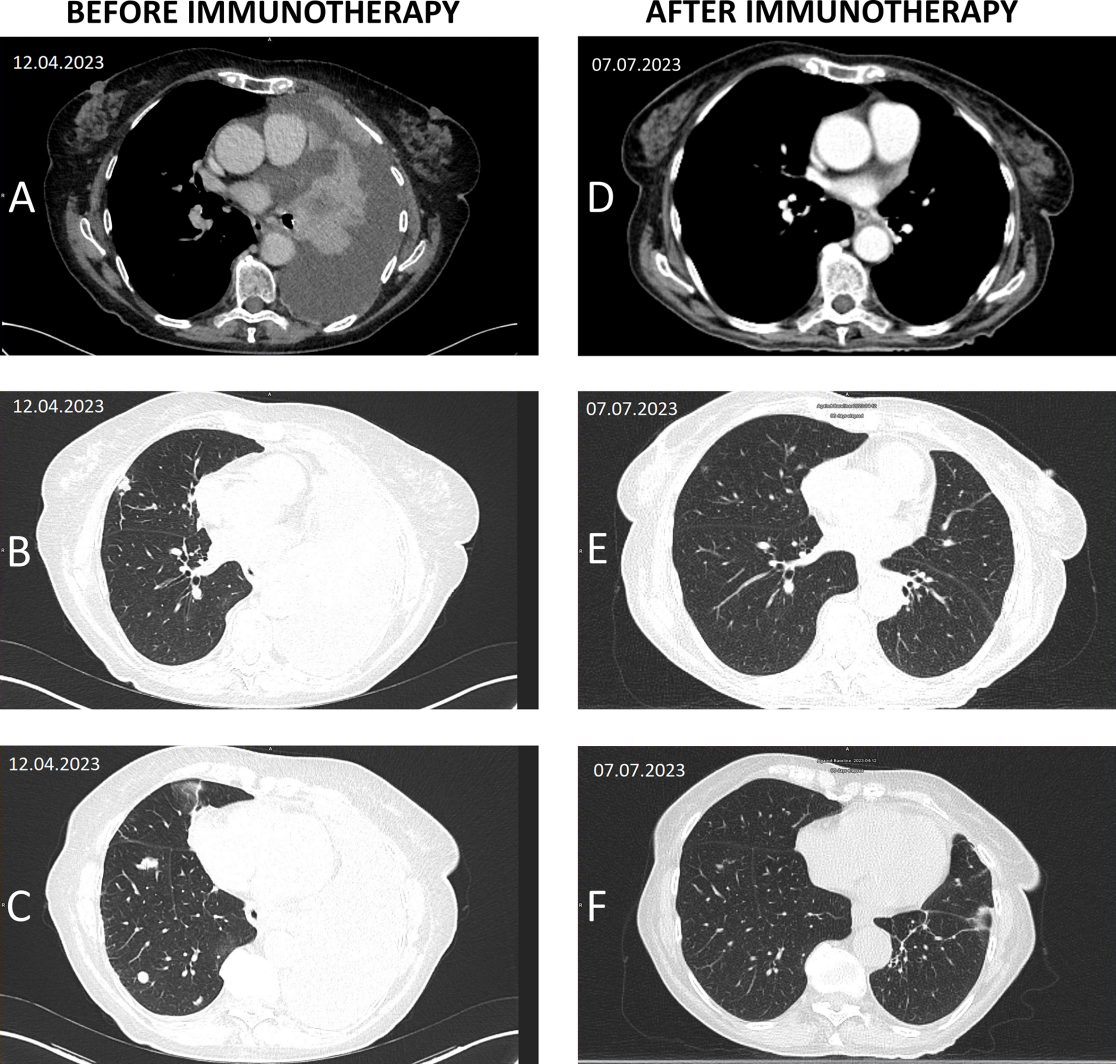
Thorax CT scan demonstrating severe left-sided pleural effusion [**(A)**, soft tissue window] and multiple ATC lung metastases [**(B, C)**, lung window]. Follow-up thorax CT examination performed three months after initiation of ICI treatment showing complete regression of metastatic lesions and pleural effusion **(D–F)**.

Two months after completing radiotherapy, the patient reported increasing fatigue, dyspnea, and a decline in performance status (ECOG 3, Eastern Cooperative Oncology Group). A chest CT performed in April 2023 revealed multiple lung metastases and severe left-sided pleural effusion (**Figures 3A–C**).

**Figure 4:**
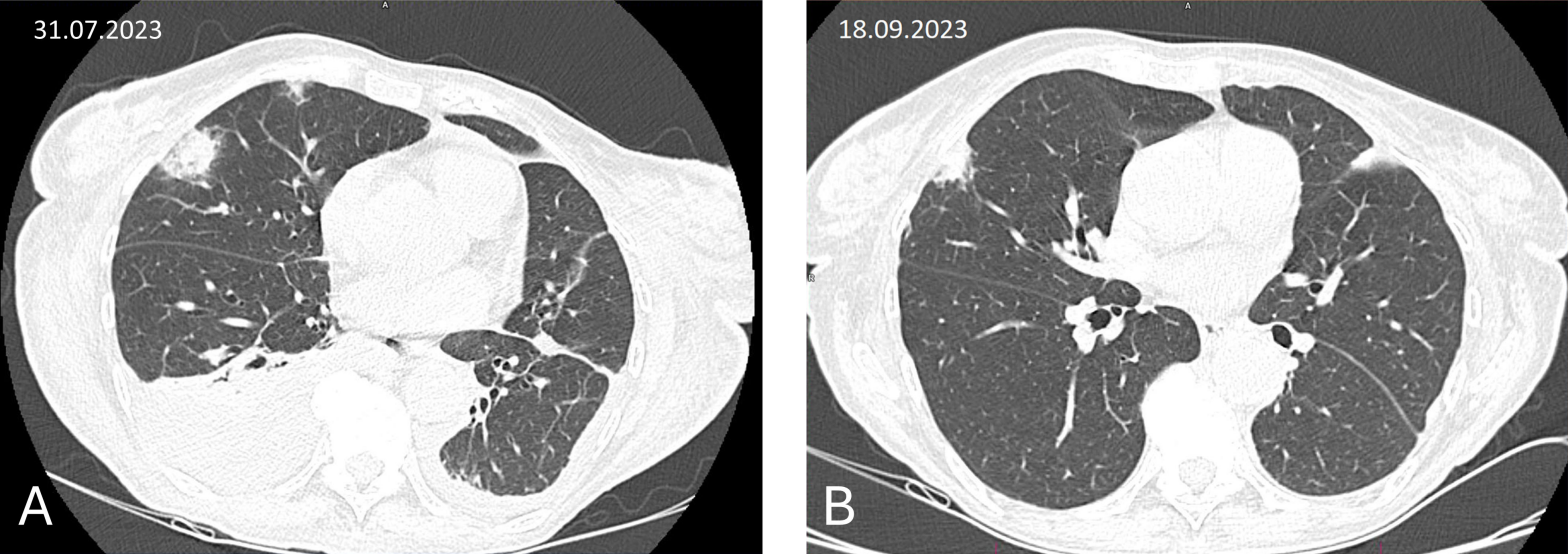
Thorax CT examination showing suspicious right lung focal lesions and bilateral pleural effusion, manifested more severely on the right side **(A)**. Follow-up thorax CT presenting with partial response of the observed right lung lesions (reduction in size) and complete resolution of pleural effusion despite the discontinuation of ICI therapy **(B)**.

Post-operative verification of the primary tumor including next-generation sequencing (NGS) did not indicate any targetable mutations that would allow systemic targeted therapy [especially *BRAF* V600E opening the way to treatment with dabrafenib plus trametinib, as currently recommended by National Comprehensive Cancer Network (30)]. However, immunohistochemical staining revealed high expression of programmed death ligand 1 (PD-L1) on the cancer cells, with tumor proportion score (TPS) 99% and combined positive score (CPS) 100 (**Figure 2F**). On this basis, the patient was offered a systemic, off-label treatment with nivolumab monotherapy, in accordance with ‘compassionate use’ described in the art. 37 of the Declaration of Helsinki. The first dose was administered at the end of April 2023. After four infusions of 240 mg of the drug every two weeks, the patient showed remarkable improvement in performance status, with resolution of dyspnea and a complete radiographic response of pulmonary metastases and pleural effusion on follow-up CT (**Figures 3D–F**). The patient was aware of the experimental nature of the treatment and fully realized the potential side-effects. After second infusion of nivolumab she experienced gradual improvement of performance status (ECOG 1), with ability to walk after third infusion. Of note, after each infusion of nivolumab, the patient suffered from transient episodes of cognitive impairment lasting 2-3 days and manifested by disorientation, memory loss, and socially inappropriate behavior (e.g., undressing in public). No other early side effects of the treatment were reported. However, at the beginning of July 2023, nine weeks after treatment initiation, the patient developed grade 3 colitis (according to the National Cancer Institute Common Terminology Criteria for Adverse Events, version 5.0) manifested by severe diarrhea, complicated with *Clostridium difficile* infection, which was effectively treated with wide-spectrum antibiotics. At this stage, nivolumab was discontinued. One month later, the patient experienced another relapse of disease with an appearance of a few suspicious lung lesions and right pleural effusion (**Figure 4A**), which, however, spontaneously regressed after another month of follow-up (**Figure 4B**) despite the discontinuation of ICI therapy. In September 2023 the patient’s condition improved sufficiently to be discharged from hospice care and then even more to be able to travel and visit Rome (ECOG 0).

**Figure 5:**
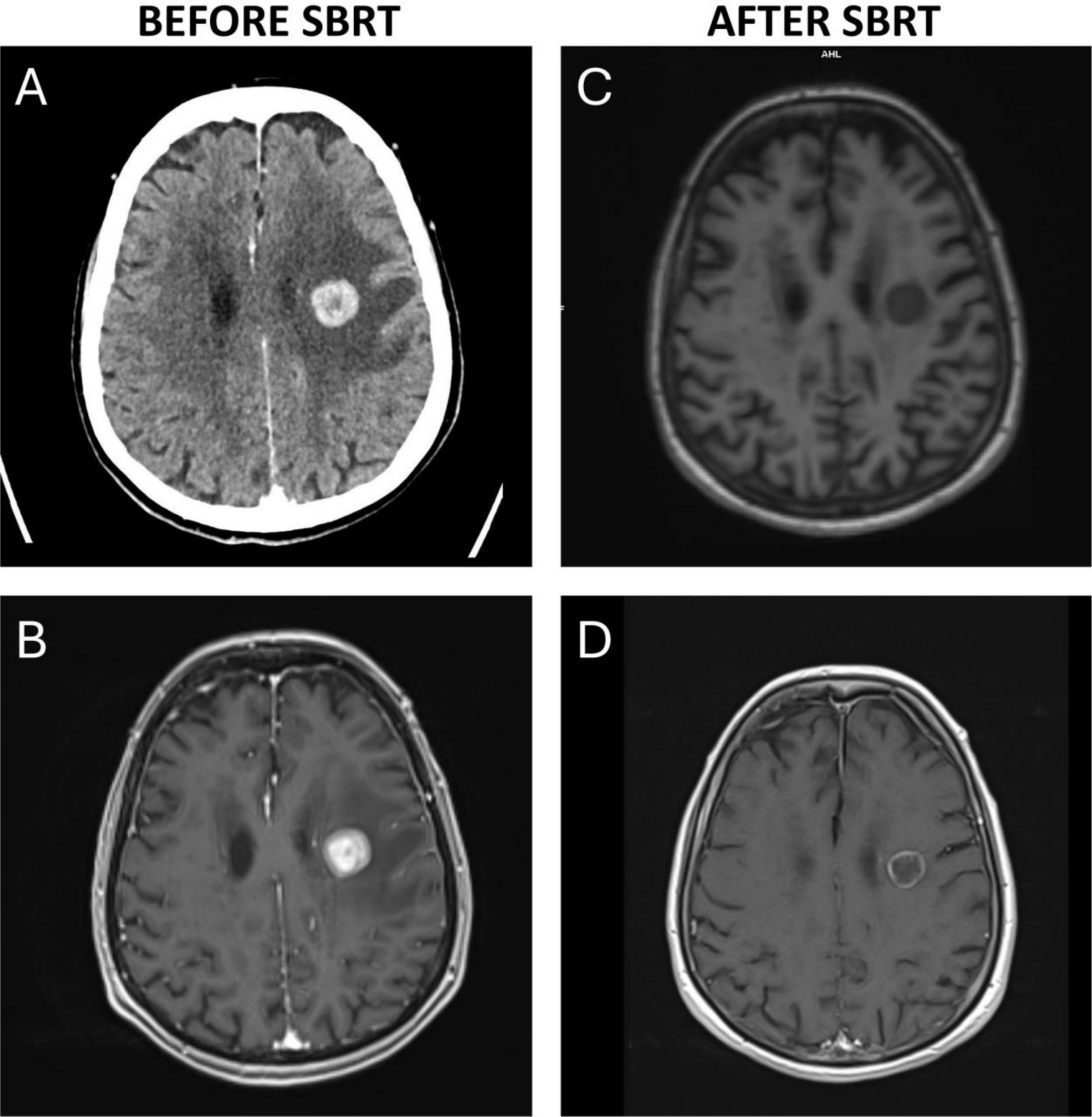
Contrast-enhanced brain CT examination [26.04.2024, **(A)**] and MRI CE-T1WI image [29.04.2024, **(B)**] demonstrating a 2 cm brain metastasis in the left frontal lobe, responsible for motoric aphasia. Follow-up MRI (09.08.2024), native T1WI **(C)** and CE-T1WI **(D)** revealed regression of peritumoral edema, in course of SBRT.

In April 2024, twelve months after beginning ICI treatment and following nine months of event-free survival, the patient developed motor aphasia. Radiographic examination revealed a single 2 cm brain metastasis in the left frontal lobe (**Figures 5A, B**). Consequently, stereotactic radiotherapy of the central nervous system lesion was performed in May 2024. The patient received a total dose of 27 Gy in 3 fractions to a PTV of approximately 6.3 cm³, corresponding to a solitary brain metastasis located at the left frontoparietal junction. The gross tumor volume (GTV) was expanded isotropically by 1 mm. As a result, complete resolution of aphasia was achieved (**Figures 5C, D**). Shortly afterwards, chest CT revealed a solitary metastasis in the posterior basal segment of the left lung. Subsequently, in June 2024, the lung lesion was managed with stereotactic body radiotherapy (SBRT) delivered in breath-hold technique. The treatment was administered to the PTV encompassing the gross tumor volume (GTV) of the left lower lobe lung lesion with an isotropic 5 mm margin. The total prescribed dose was 50 Gy in 5 fractions. In August 2024, follow-up CT revealed disease progression, with more than 20 liver metastases (up to 3 cm in diameter) and fewer than 10 new pulmonary metastases (up to 1 cm) in both lungs. Therefore, weekly metronomic palliative chemotherapy with carboplatin (AUC 1.5) and paclitaxel (80 mg/m², administered at 75% dose on days 1, 8 and 15) was offered to the patient. Despite the treatment, she experienced gradual deterioration in performance status. Eventually, the patient succumbed in October 2024 – 23 months after the primary diagnosis of ATC and 18 months after the beginning of ICI therapy.

## Discussion. Review of the literature

In recent years, ICIs have emerged as a promising therapeutic approach in the treatment of ATC, particularly in patients exhibiting high PD-L1 expression. Given the historically dismal prognosis of ATC and the limited efficacy of conventional therapies, ICIs represent a potentially transformative addition to the therapeutic arsenal.

One of the earliest pivotal trials evaluating immunotherapy in ATC was conducted by Capdevila et al. (1) who tested spartalizumab in 42 patients. The study reported an overall response rate (ORR) of 19%, with a notably higher response (35%) in tumors expressing PD-L1 ≥50% and 0% in PD-L1 negative cases. Median progression-free survival (PFS) was only 1.7 months, while median overall survival (OS) reached 5.9 months. Importantly, 24% (10 out of 42) of patients experienced immune-related adverse events (irAEs), including diarrhea and pruritus, mostly of grade 1-2 severity.

In contrast, combinations of ICIs with anti-angiogenic agents or kinase inhibitors demonstrated more substantial clinical benefits. For instance, Dierks et al. (2) reported an impressive ORR of 66% with the combination of lenvatinib and pembrolizumab in a small cohort of 6 ATC patients (4/6 PR assessed at 3-4 months and 4/6 CR 16 months after treatment), with a median PFS of 16.5 months and treatment duration ranging from 1 to 40 months. The combination was associated with a 33% rate of grade 3-4 toxicities, including anorexia and fatigue.

A retrospective cohort analysis by Hamidi et al. (3) evaluated the addition of pembrolizumab to dabrafenib and trametinib in 48 patients with *BRAF*-mutated ATC. The median OS reached 17 months, and the ORR was 73.3% among those treated with the three-drug combination. Approximately one-third of patients experienced irAEs, with hepatitis and colitis being the most frequently reported.

Dual ICI therapy has also been investigated. Sehgal et al. (4) studied the combination of nivolumab and ipilimumab in 10 patients, reporting an ORR of 30%, a clinical benefit rate (CBR) of 50%, and a median PFS of 4.3 months. This regimen, however, showed a higher incidence of grade 3 adverse events (40%), including: colitis, diarrhea, elevated serum amylase, and intracranial hemorrhage.

In one of the largest meta-analyses of therapeutic strategies for ATC, comparing various kinase inhibitor regimens with or without ICI, Tunio et al. (5) examined data from 980 patients across 47 studies. The analysis showed that ICI monotherapy had limited efficacy, with ORRs of around 16%. In contrast, combination regimens, especially those involving kinase inhibitors, achieved improved clinical outcomes; for example, patients treated with lenvatinib with pembrolizumab achieved ORR of 42% and median OS up to 14.4 months. The most common grade ≥3 toxicities included lipase and amylase elevation, adrenal insufficiency, and dermatitis. Altogether, these findings suggest that ICIs, especially in combination with targeted therapies, can produce significant and durable responses in a subset of ATC patients, most notably in those with high PD-L1 expression. Nevertheless, the risk of severe irAEs remains a significant consideration, and prospective clinical trials are needed to identify optimal therapeutic combinations and patient selection strategies.

The case presented here reflects findings reported in the literature and reinforces the potential of ICIs, particularly in PD-L1-positive ATC. Despite lacking targetable mutations such as *BRAF* V600E, the patient experienced a rapid and complete radiographic response following initiation of nivolumab, an anti-PD-1 agent. The response was consistent with other reports, especially those involving high PD-L1 expression, however to the best of our knowledge this is the only case showing complete radiographic response to nivolumab monotherapy.

While the initial response in our patient was remarkable, after 12 months of follow-up the patient experienced disease relapse and eventual progression, which is commonly described in ATC treated with ICIs, emphasizing the need for continued monitoring and possibly combination or sequential therapies to prolong response durability. However, continuing ICI treatment may require more efficient management of irAEs, including not only non-specific anti-inflammatory treatment with systemic steroids, but also specific targeted therapy of certain adverse events e.g. colitis treated with vedolizumab (31) or infliximab (32), and oral budesonide, which has recently been considered a promising option with a relatively low risk of systemic side effects (33).

The episodes of cognitive impairment which happened to our patient could have been related to the ICI treatment and deserve a short commentary. Although neurologic irAEs are relatively rare, with the estimated incidence of approximately 1-12% (34), they are difficult to classify unambiguously and can be potentially very dangerous. Most of all, they require special diagnostic vigilance and exclusion of brain infection, metastases or metabolic toxicity. In severe cases neurologic irAEs need to be treated with steroids or other immune suppression and/or discontinuation of ICI.

Overall, this case supports the inclusion of immunotherapy in the therapeutic armamentarium against ATC and highlights the importance of PD-L1 testing. It further underscores the urgent need for clinical trials and real-world data to refine treatment strategies, determine optimal combinations, and better manage adverse effects.

## Conclusions

ICIs may be a valuable therapeutic option in patients with metastatic ATC exhibiting high PD-L1 expression. The presented case report and literature review highlight the need for clinical trials to fully establish the efficacy of immunotherapy in ATC.

## Data Availability

The datasets presented in this article are not readily available because Dataset of case reports or case series of fewer than three individuals are not applicable for sharing. Any other requests should be directed to the corresponding author: Wojciech Cytawa, wcytawa@gumed.edu.pl.
