# Supplementary material for: Complete radiographic response to immunotherapy in a patient with metastatic anaplastic thyroid cancer – a case report and review of the literature
Source: Front Oncol. 2026 Jun 2;16:1780838. doi: 10.3389/fonc.2026.1780838 (PMC13269043; doi:10.3389/fonc.2026.1780838)
Supplement: Supplementary file 1 [file Table1.docx]

**Supplementary material**

Table 1 Review of the studies involving ICI therapy of ATC.

| Sample size | Age | Gender | Medication | Dose | Duration of treatment | Outcome | Complications | Anti-BRAF treatment | Initial stage | Previous therapy | Reference |
| --- | --- | --- | --- | --- | --- | --- | --- | --- | --- | --- | --- |
| 42 | Median 62.5 (range 46-83) | Male: 23 (55%), female: 19 (45%) | Spartalizumab | 400 mg IV infusion every 4 weeks | Median: **8 weeks** (range: 1.7 to 113.6 weeks); 11 patients received treatment for ≥50 weeks | - **ORR**: 19% (3 complete responses, 5 partial responses)  - **Median PFS**: 1.7 months  - **1-year OS**: 40%  - **Median OS**: 5.9 months  - Responses were **more common in PD-L1 positive tumors**, especially with PD-L1 ≥50% (ORR: 35%) | **- 45% (19/42) had treatment-related adverse events**  - Most common: diarrhea (12%), pruritus (12%), fatigue (7%), pyrexia (7%)  - irAEs in 10 patients (24%), mostly Grade 1-2 | 12 was *BRAF*-mutated | N/A | Surgery: 28, RT: 30 | (2)  Capdevila J 2020 |
| 8  (ATC: 6,  PDTC: 8)  Data in the study refer to total cohort (ATC  + PDTC) | Median 63.5 (range 49-88) | Male: 4 (50%), female: 4 (50%) | Lenvatinib + pembrolizumab | - **Lenvatinib**: 24 mg/day (adjusted by weight & tolerance); initial dose range: 14-24 mg/day  - **Pembrolizumab**: 200 mg IV every 3 weeks (after a median of 2.7 weeks after starting lenvatinib) | Range: **1-40 months**  3 patients had >24 months treatment; pembrolizumab continued after CR | - **ORR**: 75% (6/8) at 3-4 months  - **CR in 4/6 ATC patients** (66%)  - **Median OS**: 19 months (ATC only: 17.3 months)  - **Median PFS:** 17.6 months (ATC only: 16.8 months) | - **Grade 3-4 in 3/8 patients**  - Most common: hypertension (5/8), anorexia, fatigue, mucositis  - 1 grade 4 fatal bleeding (after removal of tracheostomy) | 0 | IVC | Surgery: 8,  CRT: 7, chemotherapy: 6, RAI: 2 | (3)  Dierks C 2021 |
| 48 | Median  67 (range 46–81) | Male:  26 (54%),  female: 22 (46%) | Pembrolizumab with dabrafenib/  trametinib  (pembrolizumab added to dabrafenib/trametinib before or after surgery; upfront or at progression) | N/A | Median: 10 months for dabrafenib/trametinib (range 2-63); **5 months** for pembrolizumab (range 1-55) | - **Median OS**: 17.0 months [CI, 11.9-22.1]  - The 12- and 24-month Kaplan–Meier estimates of survival of  60.2% and 36.5% | - No grade 5 events reported,  - irAEs occurred in 32%,  - Most frequent irAES: hepatitis (9%), colitis (6%), and nephritis (4%) | 100% *BRAF*-mutated | IVB 25 - 35%  IVC 65-75% | dabrafenib + trametinib for min. 2 weeks | (4)  Hamidi S 2024 |
| 10  (ATC sub-cohort) | Mean (SD): 61.4 (13.3) years | Male: 4  Female 6 | Nivolumab + ipilimumab | - Nivolumab: 3 mg/kg IV every 2 weeks  - Ipilimumab: 1 mg/kg IV every 6 weeks | Median: **135.5 days** (~4.5 months), range: 10–735 days | **ORR**: 30% (3/10 partial responses)  **CBR**: 50% (3 PR + 2 SD)  Median PFS: 4.3 months  Median OS: Not reached  24-mo OS: 55.6% (20.4-80.5)  PR: 3, SD: 2, PD: 4, CR: 0.  Unevaluable: 1 | Grade 3 AEs:  Colitis: 1,  Diarrhea: 1,  ↑ serum amylase: 1,  Intracranial hemorrhage: 1. | 3/10 had BRAF V600E, no TKI treatment while on ICI | N/A | Surgery: 9,  RAI: 3,  RT: 8,  Systemic therapy: 7. | (5)  Sehgal K 2024 |
| 71 (subset of meta-analysis of 980 patients) | N/A | N/A | Lenvatinib + pembrolizumab | N/A | Median OS 14.4 months (95% CI: 7.6–21.1 months)  Median PFS 10.0 months (95% CI: 5.3–14.6 months) | Pooled ORR 42% (95% CI, 28.6–55.3%)  Pooled DCR 64.2% (95% CI, 48.0–78.0%) | N/A | N/A | N/A | N/A | (6)  Tunio MA 2025 |
| 12 | 60 years (range 47–84 years) | Male: 8 (67%)  Female: 4 (33%) | Pembrolizumab + TKI | Pembrolizumab 200 mg IV every 3 weeks. | Median: 5.6 months (range  2.9-15.8 months) on combined therapy. | **Best overall response**: - PR: 5/12 (42%),  - SD: 4/12 (33%),  - PD: 3/12 (25%).  **Median OS** from pembrolizumab addition: 6.93 months (range 3-15.9 months).  **3 patients with OS >12 months**. | Grade 2 irAEs: 2/12 patients:  1 colitis, 1 hepatitis. | 50% had the mutation and was treated with TKI | IVB 3 (25%), IVC 9 (75%) | Surgery: 5 (41%), RT/chemosensitizing: 6 (50%),  Bridging chemotherapy: 3 (25%) | (11)  Iyer PC 2018 |
| 18 | 66 years (range: 44–80) | Female: 8 (44%),  Male: 10 (56%) | Vemurafenib + cobimetinib + atezolizumab | Not specified | Not specified | **Median OS**: 43.24 months  **Median PFS**: 13.93 months  **ORR**: 50% | Colonic perforation (1 death)  Colitis (grade 3)  Papilledema, retinopathy, pancreatitis (grade 2) | BRAF variant and treatment in all 18 | IVA: 1, IVB: 3, IVC:14 | RT 1,  CRT 2,  bridging chemotherapy 2. | (12)  COHORT 1  Cabanillas ME 2024 |
| 21 | 66 years (range: 46–83) | Female: 12 (57%), Male: 9 (43%) | Cobimetinib + atezolizumab | Not specified | Not specified | **Median OS**: 8.74 months  **Median PFS**: 4.80 months  **ORR**: 14% | Colonic perforation (grade 2)  Colitis (grade 1)  Left ventricular dysfunction (2 cases, grade 3)  Pneumonitis (grade 2) | 0 | IVA: 0, IVB: 7, IVC:14 | RT 4,  CRT 12,  bridging chemotherapy 1 | (12)  COHORT 2 |
| 3 | 51 years (range: 47–69) | Female: 2 (67%), Male: 1 (33%) | Bevacizumab + atezolizumab | Not specified | Not specified | **Median OS**: 6.21 months  **Median PFS**: 1.35 months  **ORR**: 33% | Esophageal perforation (grade 2) | 0 | IVA 0, IVB 1, IVC 2 | CRT 2,  bridging chemotherapy 1 | (12)  COHORT 3 |
| 18 | 66 (41–86) | Male: 7 (38.9%)  Female: 11 (61.1) | +*BRAF V600E* mutation:  dabrafenib + trametinib + 1/3 PD-1 inhibitors (pembrolizumab, sintilimab, or camrelizumab (physician choice)  - *BRAFV600E* mutation: oral lenvatinib or anlotinib (physician’s choice) + One of three kinds of PD-1 inhibitors (pembrolizumab, sintilimab, or camrelizumab (physician’s choice) | Dabrafenib (150mg p.o.,b.i.d.) plus trametinib (2mg p.o.,daily),  lenvatinib (24mg daily),  anlotinib (12mg daily, 2 weeks on/1 week off)  ICI: 200 mg every 3 weeks | 2 years | Median OS: 14.0 months, with the 12-month survival rate 55.6%.  ORR 61.1%:  CR: 5 (27.8%)  PR: 6 (33.3%),  SD: 1 (5.6%),  PD 3 (16.7%).  Surgical resection feasible in 7/18 (38.9%) after tumor shrinkage  Treatment 2021-2023. At the last follow up in 2024 9/18 were still alive. | One grade 5 AE occurred after initiation of lenvatinib + pembrolizumab.  Grade 3/4: 5 patients, mostly well tolerated. | 9 *BRAF* (+), 9 given dabrafenib and trametinib; *BRAF* (-) with lenvatinib or anlotinib as adjunctive treatment | IVB 8, IVC 10 | Previous surgery: 8 | (13)  Song Y 2024 |
| 5 | Median: 64 (51-74) | Male: 1 (20%)  Female: 4 (80%) | Pembrolizumab + hypofractionated RT (QUAD-shot).  Concurrent lenvatinib allowed – administered in 2 patients. | Pembrolizumab 200 mg IV every 3-4 weeks.  Twice daily 3.5 Gy (>6 h apart) for 2 consecutive days. | Pembrolizumab continued until PD or up till 24 months (treatment duration range: 15-104 weeks).  RT every 3-4 weeks between ICI cycles (total dose 56 Gy), up to 4 cycles, depending on tumor control/tolerance. | At median follow-up (32.6 months, IQR: 26.4–38.8):  **ORR**: 80% with **CR**: 2 and **PR**: 2.  CR patients disease-free at last follow-up, despite discontinuation of treatment.  **Median PFS**: 7.6 months (IQR: 6.2–not reached).  1-year PFS/OS: 40% (95% CI: 13.7–100) each. | Mostly grade 1 and 2 AEs. | 1 *BRAF* V600E mutated (refused targeted therapy) | N/A | Surgery: 2 | (14)  Tan JSH 2024 |
| 1 | 53 | Male | Pembrolizumab | 200 mg | 4 doses over ~2 months | - Initial PR: 53% total tumor burden reduction; liver metastases decreased on follow-up.  - Then PD: brain metastases on CT 3 months post-ICI, metastatic bowel perforation at 5 months post-ICI, deceased 1 month later.  OS 9 months | N/A | *BRAF* V600E negative | IVC | CRT | (15)  Aghajani MJ 2019 |
| 2 | 1: 55  2: 59 | **Patient 1**: male  **Patient 2**: female | **Patient 1:** carboplatin and paclitaxel + pembrolizumab with  lenvatinib  **Patient 2:** Lenvatinib, pembrolizumab | **Patient 1:** carboplatin AUC5 and paclitaxel 175 mg/m2 (for 1 cycle as a temporizing measure)  **Patient 2:** pembrolizumab 200 mg every 3 weeks and lenvatinib 14 mg daily | **Patient 1:** 2 cycles (5 weeks)  **Patient 2:** 12 weeks; still on  therapy when completing the publication | **Patient 1:** Initially significant reduction in activity and size on PET, deceased 5 months after diagnosis  **Patient 2:** early treatment response in neck on CT scans | **Patient 1**: Pure red cell aplasia complicating pembrolizumab, discontinuation of ICI | Both was *BRAF* negative | N/A | **Patient 1**: surgery,  **Patient 2**: none | (16)  McCrary HC 2022 |
| 5 | 65 (52–72) | Male: 3  Female: 2 | Lenvatinib and pembrolizumab | Lenvatinib starting dose 14 to 24 mg daily and pembrolizumab 200 mg every 3 weeks | N/A | The median PFS was 4.7 (range 0.8-5.9) months, and the median OS was 6.3 (range 0.8-not reached) months  SD: 3; PR: 1; not evaluable: 1 | AEs seen in all patients, in 2 discontinuation of therapy was necessary | 2 with *BRAF V600E* | IVB 1, IVC 4 | All 5 underwent previous surgery | (17)  Soll D 2024 |
| 2 | 1: 59  2: 73 | **Patient 1**: Male  **Patient 2**:  Female | **Patient 1:** Pembrolizumab/ axitinib  Dabrafenib/ trametinib  Lenvatinib  **Patient 2:** Docetaxel/doxorubicin plus  Pembrolizumab | N/A | **Patient 1:** 15 months  (pembrolizumab started 3 months after initiation of pemetrexed + carboplatin)  **Patient 2:**  2.5 months then stopped due to unsatisfactory results | **Patient 1**: RECIST response: -54% **PR**  PFS 13.8 months  **Patient 2**:  +18% PD  PFS 2.4 | N/A | **Patient 1:**  *BRAF* +  **Patient 2:**  *BRAF* - | **Patient 1**: IVB  **Patient 2**: loco- regional | **Patient 1**: 2 lines of systemic treatment  **Patient 2**: 1 line of systemic treatment | (18)  Lee KK 2025 |
| 1 | 65 | Male | Initially nivolumab + TKI (cabozantinib),  later nivolumab monotherapy. | Nivolumab, initially 240 mg every 2 weeks, later 200 mg every 3 weeks.  Cabozantinib, 40 mg (later discontinued due to thrombotic events). | 2 years | PR:  - radiographic response: reduction in size of brain and adrenal metastases,  - clinical benefit in >30 months follow-up. | AEs (combined irAEs and TKI-related AEs):  - gastroenteritis,  - pneumonia,  both maintained below grade 1 with steroid treatment. | N/A | N/A | Surgery, CRT, TKI monotherapy | (20)  Ma DX 2022 |
| 1 | 60 | Male | Dabrafenib + trametinib  + pembrolizumab  + surgery  + CRT | Dabrafenib, 150 mg PO twice daily.  Trametinib, 2 mg daily.  Pembrolizumab, 200 mg IV every 3 weeks.  Post-operative RT: 57-60 Gy/30 fractions (residual disease) over 6 weeks with concurrent radiosensitizing chemotherapy (cisplatin) + pembrolizumab. | N/A | On DTP (dabrafenib + trametinib + pembrolizumab) therapy:  **PR** - the tumor became resectable.  After surgery and RT (continued DTP):  **CR** - resolution of neck lymph nodes and dermal metastases.  No evidence of disease 16 months after diagnosis. | N/A | *BRAF*-mutated, targeted therapy: dabrafenib + trametinib | N/A | Chemotherapy, TKI. | (19)  Cabanillas ME 2018 |
| 1 | 69 | Male | Pembrolizumab | 200 mg IV every 3 weeks | 25 doses (continued) | Significant clinical improvement at 1 week after initiation of treatment.  PR after 4 treatment cycles.  No progression at 18 months after diagnosis. | well tolerated irAE - thyroiditis (persistent hypothyroidism) | *BRAF* V600E negative | N/A | None | (20)  Nabhan F 2021 |
| 3 | Median age: 56 | Male: 1  Female: 2 | Pembrolizumab + CRT (docetaxel/ doxorubicin) + volumetric modulated arc therapy (VMAT) | Pembrolizumab, 200 mg IV every 3 weeks.  Docetaxel/doxorubicin, 20 mg/m2 each IV weekly (dose reduced by 25% in patient 2 protocol due to adverse effects)  VMAT: 66 Gy/33 fractions (gross disease) + 59.4 Gy/33 fractions (elective lymph nodes) over 6.5 weeks. | Patient 1: 4 cycles  Patient 2 and 3: 3 cycles of pembrolizumab. | Study terminated early because none of the patients reached the primary endpoint of 6 months median OS. | Multiple AEs, at least possible immune-related:  - grade 5: pneumonitis and respiratory failure,  - lesser grades: sepsis, laryngeal edema, lung infection, nausea, vomiting | 1 was *BRAF*-mutated, no targeted treatment administered | IVB 3 | 2 has previous surgery | (21)  Chintakuntlawar A V. 2019 |
| 1 | 47 | Female | RT + tislelizumab | Initially, 4500 cGy/15 fractions (neck) + 6750 cGy/15 fractions (tumor boost).  Then: tislelizumab, 200 mg every 3 weeks. | N/A (≥ 15 months) | PR | Almost none | *BRAF V600E* negative | N/A | Surgery + RAI performed years earlier for previously diagnosed PTC | (22)  Xing Y 2023 |
| 1 | 59 | Female | Tislelizumab | Tislelizumab, 200 mg every 3 weeks.  Initially combined with nab-paclitaxel (125 mg/m² on days 1 and 8, planned for every 3 weeks) - discontinued after 1^st^ cycle due to intolerance. | 14 months and continued for maintenance treatment | 14 months of sustained remission.  PR at ~6 weeks; CR at ~3.5 months from ICI initiation. | None | *BRAF V600E* negative | N/A | Surgery, RT, TKI | (23)  Chai J 2025 |
| 1 | 75 | Female | Pembrolizumab | N/A | 8 cycles of pembrolizumab - discontinued  because of grade 4 gastro-intestinal irAE. | After 2 cycles clear clinical response, after 3 cycles almost CR on CT.  No signs of progression 3 months after the last dose of pembrolizumab.  Patient died 18 months after initial diagnosis. | After 8 cycles severe colitis (grade 4) with bloody stools, treated with steroids and infliximab. | N/A | IVC | None | (8)  Spalart V 2019 |
| 1 | 37 | Male | Initially 10 months of 1^st^ line chemotherapy, then:  gemcitabine + **toripalimab** (the PD-1 inhibitor was discontinued after 1^st^ dose due to irAE).  Then: gemcitabine + S-1 (3 cycles followed by one S-1 cycle).  Disease progression after 9 months: **zimberelimab** + nab-paclitaxel. | gemcitabine 1.6 g + **toripalimab 240 mg** (single dose)  **zimberelimab 240 mg**, plus nab-paclitaxel 200 mg IV  every 2 weeks | Total 9 cycles of zimberelimab and nab-paclitaxel combined then single zimberelimab for maintenance therapy. | After 3 cycles of zimberelimab + nab-paclitaxel regression of both thyroid and liver lesions leading to PR (best overall response) and remained stable for 34 months (the last check-up). | Toripalimab: rash (grade 3) and hepatitis (grade 1).  Zimberelimab: hypothyroiditis (grade 2). | N/A | IVC | Chemotherapy | (24)  Fan S 2025 |
| 1 | 71 | Male | Pembrolizumab + lenvatinib | Pembrolizumab 50 mg x2 doses + lenvatinib 10/20 mg/day oral (alternatively) | 1 month | No tumor shrinkage, death from hypoxic respiratory failure | Dyspnea, dizziness, headache, pneumonitis-like changes, respiratory failure | no treatment | IVB | Surgery | (25)  Shih SR 2022 |
| 1 | 59 | Male | Spartalizumab | 400 mg IV every 4 weeks | Ongoing (≥45.5 months) | SD >23 months; alive at 45.5 months | Deep neck infection at 16 months, pseudoprogression, dysphagia | no treatment | IVC | Surgery | (25) |
| 1 | 60 | Female | Pembrolizumab + lenvatinib (later dabrafenib + trametinib) | Pembrolizumab 200 mg IV + lenvatinib titrated to 24 mg/day oral; then dabrafenib 300 mg + trametinib 2 mg daily | 11.4 months | Initial response, then progression; death after 18 months | Grade 4 hepatitis, pneumonitis, tracheoesophageal fistula, cholestatic hepatitis, respiratory failure | *BRAF* V600E-mutated with targeted treatment | IVC | Surgery | (25) |
| 1 | 81 | Female | Neoadjuvant:  lenvatinib  Surgery (×2: thyroidectomy and pulmonary metastasectomy)  Adjuvant: lenvatinib  + **pembrolizumab**  RT after discontinuation of ICI treatment. | Neoadjuvant:  lenvatinib 20 mg daily  Adjuvant: lenvatinib dose as above + **pembrolizumab** dose not reported | Lenvatinib 26 days pre-op + resumed 14 days post-op.  **Pembrolizumab** duration not reported. | **PD**: local recurrence in the thyroid bed at 4 months post-surgery,  died 8 months from diagnosis | irAEs:  allergic dermatitis and skin detachment – led to discontinuation of ICI-treatment | *BRAF V600E* negative | IVC | No treatment | (26)  Maurer E 2023 |
| 1 | 57 | Male | Neoadjuvant:  lenvatinib + **pembrolizumab**  Surgery  Adjuvant: lenvatinib + **pembrolizumab**  Chemotherapy (carboplatin + paclitaxel) after discontinuation of mKI/ICI treatment. | Neoadjuvant:  lenvatinib 20 mg daily + **pembrolizumab** 200 mg on day 1 and 22  Adjuvant: lenvatinib + **pembrolizumab** doses as above | Lenvatinib 32 days pre-op + resumed 6 days post-op  **Pembrolizumab** 2 doses pre-op + resumed 10 days post-op and continued every 3 weeks.  Both medications administered for 4 months post-op. | **SD** at 11 months post-surgery | AEs: extensive colitis – led to discontinuation of mKI/ICI-treatment after 4 months | *BRAF V600E* negative | IVC | No treatment | (26) |
| 1 | 73 | Male | Neoadjuvant:  lenvatinib + dabrafenib + trametinib  Surgery  Neoadjuvant: dabrafenib + trametinib, then switched to lenvatinib + **pembrolizumab** | Neoadjuvant:  lenvatinib 20 mg daily switched to  dabrafenib 150 mg twice daily (reduced to 75 mg twice daily due to side effects) + trametinib 4 mg daily after 4 weeks  Adjuvant:  The BRAF-targeted treatment continued resumed post-op, then switched to lenvatinib + **pembrolizumab** (doses not reported) | Neoadjuvant treatment for 43 days pre-op.  Adjuvant treatment: dabrafenib + trametinib resumed 10 days post op and continued, switched to lenvatinib + pembrolizumab at recurrence 6 months later. | **SD** at 8 months post-surgery | AEs (BRAF-targeted therapy-related): leucopenia and fever, dose adjustment needed. | BRAF-mutant, targeted treatment: dabrafenib + trametinib | IVB | No treatment | (26) |
| 1 | 63 | Female | RT + pembrolizumab | Pembrolizumab, 2 mg/kg every 3 weeks.  RT, 50-75 Gy/25 fractions. | 10 cycles of pembrolizumab over ~8 months | **CR** maintained at 26-month follow-up. | Grade 1 irAEs: hepatitis and adrenal insufficiency. | N/A | IVB | Surgery + RAI performed for previously diagnosed PTC | (27)  Yang SR 2021 |
| 1 | 51 | Female | Pembrolizumab + RT | Pembrolizumab 4-weekly (dose not specified).  QUADshot hypofractioned RT: 30 Gy | Pembrolizumab continued for 2 years | **CR** after 2 cycles of pembrolizumab + RT, sustained for 32 months (local and distant: lung, suprahilar node).  Later: a single bone metastasis. | No long-term irAEs during 2-year pembrolizumab treatment | *BRAF* V600E negative | N/A | Surgery | (28)  Goh D 2023 |
| 1 | 49 | Male | Pembrolizumab | 3 cycles | N/A | **PD**: diffuse bone + new liver metastases | N/A | N/A | N/A | Surgery, CRT | (29)  Sukari A 2020 |
| 1 | 61 | Female | **Pembrolizumab** + Carboplatin + Paclitaxel  After completion if the above therapy: dabrafenib + trametinib resumed | 4 cycles | N/A | **SD** at 10 months post-ICI and 22 months from diagnosis follow-up (only a single left lung nodule reduced in size) | N/A | *BRAF* V600E mutated, targeted therapy (dabrafenib + trametinib) | IVC | Surgery, CRT, TKI | (29) |
| 12 | Median 71 (range 49–88) | Male: 6  Female: 6 | Durvalumab + tremelimumab + SBRT (9 Gy x 3) | Durvalumab 1500 mg; tremelimumab 75 mg (4 doses max) | Median 11 weeks (range 1-36 weeks) | Median OS 14.5 weeks (range 12–89); only 1 patient alive >1 year; no confirmed responses, 1 SD. | Grade 4 AE: 1 (respiratory failure).  Grade 3 AE: 9, incl. pancreatitis, dysphagia, anemia, lung infection, and others. | no treatment | IVC | Prior RT:  10 patients | (30)  Lee NY 2022 |
| 1 | 55 | Female | **Camrelizumab** + apatinib | **Camrelizumab** dose not reported.  Apatinib, 250 mg daily | 11 months and continued | Local recurrence at 6 months post-ICI (treated with RT).  Clinically stable at 11 months of ICI | No irAEs reported | N/A | IVB | Surgery,  apatinib monotherapy | (31)  Zheng L 2021 |
| 1 | 62 | Male | Nivolumab added to vemurafenib, then continued as monotherapy | Nivolumab dose not reported. | 12 cycles, then stopped due to colitis exacerbation | **CR** (radiographic and clinical) at 20 months follow-up post-ICI. | IrAEs:  - exacerbation of psoriasis – managed locally,  - grade 2 colitis (pre-existing ulcerative colitis in remission) treated with steroids + mesalamine; led to ICI discontinuation | *BRAF* V600E mutated, targeted therapy: vemurafenib | N/A | Surgery + RAI performed for previously diagnosed PTC.  For ATC: chemotherapy and anti-BRAF treatment (vemurafenib) | (32)  Kollipara R 2017 |
| 1 | 67 | Female | Sintilimab + anlotinib | Sintilimab 200 mg every 3 weeks.  Anlotinib 12 mg, once daily, 2-week on/1-week off. | 18.3 months | **PR** (prominent and sustained reduction of tumor) | Grade 1 rash | no treatment | IVB | Surgery | (33)  Gui L 2021 |
| 1 | 54 | Female | Lenvatinib + **Pembrolizumab** | Lenvatinib 24→10 mg daily.  Pembrolizumab 200 mg every 3 weeks (after 12 months: every 6 weeks). | 18 months and continued | **PR:**  - 76% reduction in dominant lung metastasis after 5 months of treatment,  - 50% reduction in size if all lung target lesions after 18 months of treatment.  Single pleural lesion progression - treated with SBRT (42 Gy in 7 fractions). | AEs considered TKI-related: grade 3 diarrhea, vomiting, and weight loss → lenvatinib dose reduction,  poor compliance with lenvatinib | *BRAF* V600E negative | IVB | Surgery, chemotherapy | (34)  Luongo C 2021 |

AEs – adverse events, ATC – anaplastic thyroid carcinoma, CBR – clinical benefit rate, CR – complete response, CRT – chemoradiotherapy, ICI – immune checkpoint inhibitor, IV – intravenous, irAE(s) – immune-related adverse event(s), N/A – not applicable, ORR – objective response rate, DCR – disease control rate, PFS – progression-free survival, OS – overall survival, PD – progressive disease, PD-1 - programmed cell death protein 1, PD-L1 – programmed cell death ligand 1, PDTC – poorly differentiated thyroid carcinoma, PO – per os; PR – partial response, PTC – papillary thyroid carcinoma, RAI – Radioactive Iodine Therapy, RECIST - Response Evaluation Criteria in Solid Tumors, RT – radiotherapy, SBRT – stereotactic body radiotherapy, SD – stable disease, TKI – thyrosine kinase inhibitor
